# Discovery and Validation of Circulating EVL mRNA as a Prognostic Biomarker in Pancreatic Cancer

**DOI:** 10.1155/2021/6656337

**Published:** 2021-04-20

**Authors:** Yan Du, Kai Yao, Qingbo Feng, Feiyu Mao, Zechang Xin, Peng Xu, Jie Yao

**Affiliations:** ^1^Clinic Medical College, Dalian Medical University, Dalian 116000, China; ^2^Clinic Medical College, Yangzhou University, Yangzhou 225000, China; ^3^Department of Liver Surgery, West China Hospital Sichuan University, Chengdu 610000, China; ^4^Department of Hepatobiliary and Pancreatic Surgery, Northern Jiangsu People's Hospital, Clinic Medical College, Yangzhou University, Yangzhou 225000, China

## Abstract

**Background:**

Circulating plasma mRNAs can be analyzed to identify putative cancer biomarkers. This study was conducted in an effort to detect plasma mRNA biomarkers capable of predicting pancreatic cancer (PACA) patient prognosis. *Material and Methods*. First, prognostic mRNAs that were differentially expressed in PACA in The Cancer Genome Atlas (TCGA) were established, after which microarray expression profiles from PACA patient plasma samples were utilized to specifically identify potential prognostic plasma mRNA biomarkers associated with this cancer type. In total, plasma samples were then collected from 79 PACA patients and 19 healthy controls to confirm differential mRNA expression via qPCR, while Kaplan–Meier analyses were used to examine the link between mRNA expression and patient overall survival.

**Results:**

In total, three prognostic differentially expressed genes were identified in PACA patient plasma samples, including SMAP2, PTPN6, and EVL (Ena/VASP-like). Plasma EVL levels were confirmed via qPCR to be correlated with tumor pathology (*p* < 0.01), while the overall survival of patients with low plasma EVL levels was poor (*p* < 0.01). Multivariate Cox regression analyses further confirmed that plasma EVL levels were independent predictors of PACA patient prognosis.

**Conclusion:**

We found that PACA is associated with the downregulation of plasma EVL mRNA levels, indicating that this mRNA may be a viable biomarker associated with patient prognosis.

## 1. Introduction

Pancreatic cancer (PACA) is among the deadliest forms of cancer globally [1], accounting for the fourth-highest rate of cancer-related mortality in the USA with a survival rate of below 9% [2, 3]. The prognosis of PACA is generally very poor in part because tumors are asymptomatic during their early stages such that they are rarely detected until after they have metastasized, at which time patients have generally poor outcomes and a 5-year survival rate of below 3% [1].

Owing to the poor outcomes associated with this disease, there is an urgent need for the identification of novel biomarkers of localized PACA that can be used to predict tumor progression and guide timely treatment efforts. Hematological biomarkers have been identified in recent years as promising candidates capable of guiding the diagnosis and monitoring of many cancers, including PACA [4–7]. For example, in one clinical study, galectin-9 was shown to be highly expressed in human PACA and to be correlated with patient prognosis through an analysis of galectin-9 levels in serum samples from 70 PACA patients, 36 patients with benign pancreatic diseases, and 28 healthy controls [8]. One database study found serum LAMC2 levels to exhibit a significantly improved diagnostic utility relative to CA199 when discriminating between PACA patients, healthy controls, and individuals with benign diseases [9]. In a separate study, researchers identified a 25-component PACA serum biomarker signature through gene expression analyses of serum samples from 34 pancreatic cancer patients and 30 healthy controls [10].

Herein, we utilized The Cancer Genome Atlas (TCGA) database and microarray sequencing analyses of clinical patient plasma samples to identify mRNAs associated with PACA patient prognosis. In total, we identified three mRNAs that were downregulated in PACA patient plasma and correlated with patient survival outcomes. We subsequently confirmed the prognostic relevance of plasma EVL mRNA expression levels in PACA patients by analyzing plasma samples from 79 PACA patients and 19 healthy controls.

## 2. Materials and Methods

### 2.1. Bioinformatics Analysis

RNA sequencing data pertaining to PACA tumor tissue samples and paracancerous control tissues were downloaded from the TCGA database (https://portal.gdc.cancer.gov/accessed August 20, 2020). Information related to patient overall survival (OS) was obtained for all patients. The DESeq R package was then used to standardize this PACA RNA transcriptomic dataset, after which R v. x64 3.6.4 and the edgeR package were used to identify differentially expressed (DE) mRNAs associated with PACA using the following screening criteria: FC > 2 and FDR < 0.05. Kaplan–Meier analyses were used to evaluate the link between mRNA expression and patient OS using the R Survival package.

### 2.2. mRNA Expression Profiling

TRIzol (Takara, Japan) was used to extract total RNA from patient plasma samples, after which human protein-coding transcripts in these samples were profiled with Affymetrix Human mRNA Array 2.0 (HTA 2.0) GeneChips (Affymetrix, CA, USA) by QiMing Biotech (Shanghai, China). Briefly, rRNA was removed from plasma samples, followed by transcription and amplification to prepare full-length fluorescent cRNAs without 3′ bias. Each cRNA was then hybridized to the Affymetrix Human mRNA Array, and sample labeling and hybridization were conducted with the Affymetrix Microarray-Based Gene Expression Analysis protocol.

### 2.3. Microarray Data Analysis

For microarray analyses, differentially expressed genes were identified using the following criteria: |FC| > 1.5, *p* < 0.05, and FDR < 0.05. Gene Ontology (GO) enrichment analyses (http://www.geneontology.org) were employed to assess the relationship between these differentially expressed genes and specific biological processes (BPs), cellular components (CCs), and molecular functions (MFs). Kyoto Encyclopedia of Genes and Genomes (KEGG, http://www.genome.jp/kegg) enrichment analyses were also conducted to establish the enrichment of these genes in specific biological pathways.

### 2.4. Sample Collection

In total, 79 PACA patients and 19 healthy controls were included in this study. Plasma mRNA samples from 7 PACA patients and 3 healthy controls were subjected to plasma mRNA array profiling, after which the observed differences in plasma mRNA expression were validated using all 79 PACA patient and control samples. These samples were collected between January 2015 and September 2019 from pancreatic ductal cell carcinoma patients undergoing postoperative pathological evaluation. Patient clinicopathological characteristics were collected, and staging was performed as per the American Joint Committee on Cancer (AJCC) criteria. The most recent patient follow-up was conducted on September 30, 2020, and patient survival was calculated from the date of surgery to the date of death or most recent follow-up. All patients provided written informed consent, and the Ethics Committee of Northern Jiangsu People's Hospital approved this study.

### 2.5. qPCR

A StepOnePlus Real-Time PCR System (Applied Biosystems, NY, USA) was used to conduct qPCR assays. Briefly, cDNA was prepared with a PrimeScript RT Reagent Kit. All qPCR reactions were conducted in a 20 ul volume containing 10 ng of cDNA based on provided instructions. Relative EVL expression was assessed via the 2^−ΔΔCt^ approach, and primers used were as follows: 5′-CTCAAAGTCCGATGCCAACC-3′ (forward) and 5′-TCTTGGCCAGCAGTTTGTTC-3′ (reverse) for EVL and 5′-CTCGCTTCGGCAGCACA-3′ (forward) and 5′-AACGCTTCACGAATTTGCGT-3′ (reverse) for U6. All qPCR analyses were conducted in triplicate.

### 2.6. Statistical Analysis

SPSS 24.0 (Chicago, IL, USA) and Prism 7 (GraphPad Software, Inc., CA, USA) were used for statistical testing. Categorical data are given as frequencies and percentages. The link between EVL expression and patient clinicopathological characteristics was assessed via a chi-squared test, while Spearman's rank correlation coefficients were used to gauge bivariate correlations. Kaplan–Meier survival curves and log-rank tests were used to assess patient survival. *p* < 0.05 was the significance threshold.

## 3. Results

### 3.1. Identification of PACA-Related DE mRNAs in the TCGA Database

**Figure 1:**
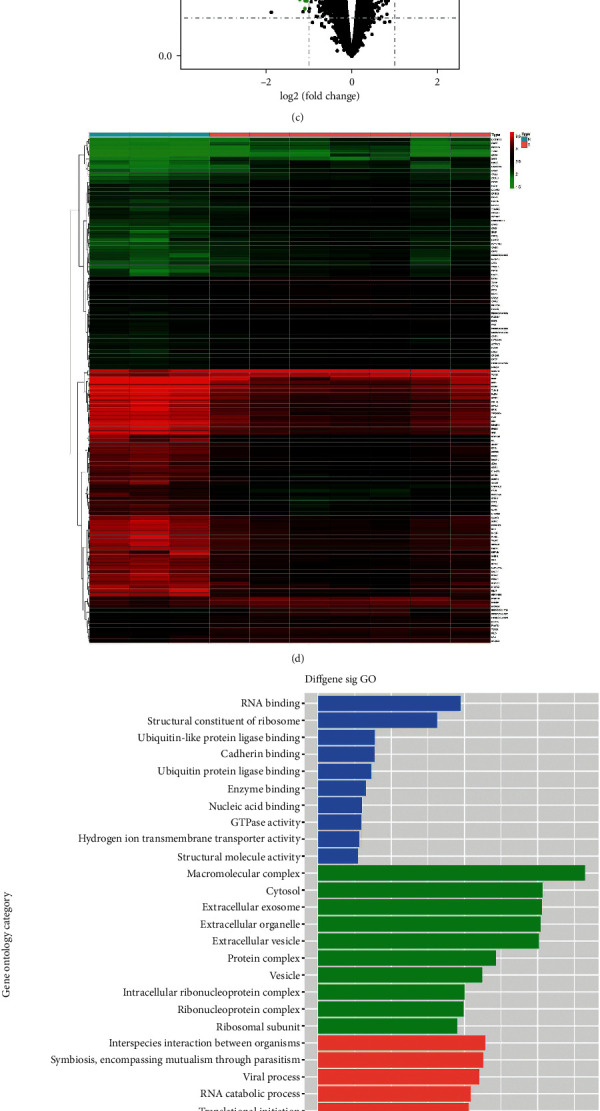
Identification of mRNAs that are differentially regulated in the TCGA database and in microarray-based plasma mRNA expression profiles from PACA patients. (a), (b) Volcano plots and hierarchical clustering analyses were used to identify mRNAs that were differentially expressed between pancreatic tumor tissue and control samples in the TCGA dataset. (c), (d) Volcano plots and hierarchical clustering analyses were used to detect mRNAs that were differentially expressed between pancreatic tumor tissue and control samples in our microarray dataset. (e) Differentially expressed mRNAs were subjected to GO enrichment analyses of key biological processes, cellular components, and molecular functions. (f) Top enriched KEGG pathways for differentially expressed mRNAs in the present microarray dataset. The size of the circle represents the number of genes enriched in the pathway. Circle colors correspond to *p* values. TCGA, The Cancer Genome Atlas; DE mRNA, differentially expressed mRNA; and KEGG, Kyoto Encyclopedia of Genes and Genomes.

We began by comparing mRNA expression profiles between PACA patient tumor and paracancerous tissue samples in the TCGA database. In total, 823 DE mRNAs were identified in PACA tumors when comparing these two tissue types (*p* < 0.05 and FC ≥ 2.0), of which 34 were upregulated and 789 were downregulated (Figure 1(a)). Hierarchical clustering analyses clearly demonstrated that we were able to differentiate between tumor and paracancerous tissue samples based upon these DE mRNA expression profiles (Figure 1(b)).

### 3.2. Identification of Prognostic DE mRNAs in Patient Tissue Samples

Next, the relationship between identified DE mRNAs and PACA patient OS was evaluated using Kaplan–Meier curves and the log-rank test based upon PACA patient survival data in the TCGA database. In total, 94 DE mRNAs were found to be correlated with PACA patient prognosis.

### 3.3. Identification of PACA-Related DE mRNAs in Patient Plasma Tissue Samples

DE mRNAs in plasma samples from PACA and control patients were next identified using a microarray-based approach. In total, this analysis led to the identification of 2240 DE mRNAs in the plasma of PACA patients (*p* < 0.05 and FC ≥ 1.5), of which 152 and 2088 were up- and downregulated, respectively (Figures 1(c) and 1(d)). GO and KEGG enrichment analyses were conducted to assess the enrichment of these DE mRNAs in specific biological pathways, compartments, and functional classifications, with the most enriched GO and KEGG terms being shown in Figures 1(e) and 1(f), respectively.

### 3.4. Identification of Prognostic DE mRNAs in Patient Plasma Samples

**Figure 2:**
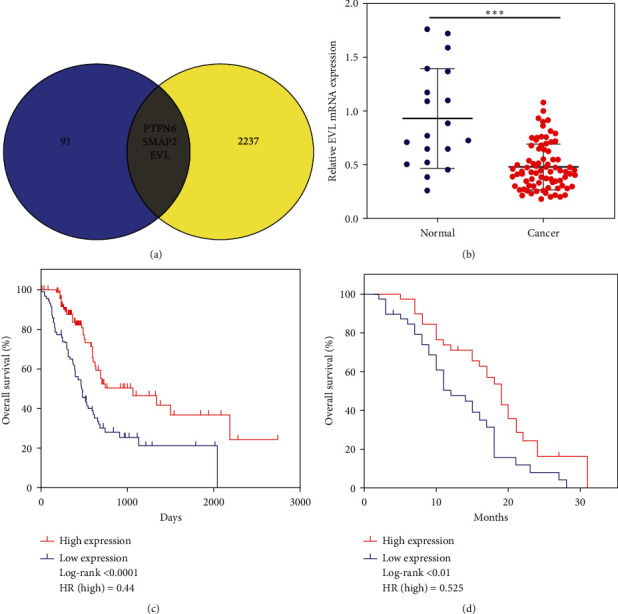
Plasma EVL levels are decreased in patients with pancreatic cancer. (a) Overlapping genes between the TCGA prognostic gene set and the differentially expressed pancreatic-cancer-associated plasma microarray gene set. (b) Plasma EVL levels were significantly decreased in plasma samples from 79 pancreatic cancer patients relative to 19 normal controls as determined via qPCR. (c), (d) Kaplan–Meier survival curves revealed that elevated EVL was associated with better overall pancreatic cancer patient survival in both the TCGA database ((c), *p* < 0.0001) and the present clinical dataset ((d), *p* < 0.01); *p* values were calculated via the log-rank test. EVL, Ena/VASP-like; qPCR, real-time quantitative polymerase chain reaction; and TCGA, The Cancer Genome Atlas.

In our TCGA analysis, we had identified 94 prognosis-related DE mRNAs. Using a Venn diagram package, we determined which of these 94 mRNAs overlapped with our list of 2240 DE mRNAs detected in PACA patient plasma samples (Figure 2(a)), ultimately leading to the identification of three prognostic DEGs in patient plasma samples: PTPN6, EVL, and SMAP2. As EVL exhibited the strongest prognostic correlation of these three genes in the TCGA patient cohort, we selected it as a target for further study.

### 3.5. EVL Downregulation Is Linked to PACA Patient Clinicopathological Features

We determined that EVL mRNA expression was significantly decreased in the majority of tested PACA patient plasma samples (*n* = 79) relative to normal control patient plasma samples (*n* = 19; *p* < 0.001; Figure 2(b)). To explore the clinical significance of EVL expression (Table 1), we next interrogated the link between its expression and PACA patient clinicopathological characteristics. We determined that EVL expression was negatively correlated with PACA pathological stage (*p* < 0.01) and patient age (*p* < 0.05), but was unrelated to patient sex, clinical stage, TNM classification, vascular invasion status, or nerve invasion status. Spearman's correlation analyses of the relationship between EVL and these parameters yielded comparable results (Table 2).

### 3.6. Decreased EVL Expression Is Predictive of Poor Prognosis

Using the TCGA database, we evaluated the prognostic significance of EVL expression levels in PACA. The Kaplan–Meier survival curve revealed that a low EVL expression level was associated with a poorer patient prognosis (*p* < 0.0001, Figure 2(c)). In order to further verify the prognostic value of plasma EVL levels, we collected follow-up data for these 79 PACA patients, and the results of survival analyses showed EVL low expression to be associated with poorer OS in plasma samples (*p* < 0.01Figure 2(d)). Univariate Cox regression analyses identified EVL expression (*p* < 0.01), *T* classification (*p* < 0.01), *M* classification (*p* < 0.01), and nerve invasion (*p* < 0.05) as predictors of poorer PACA patient OS. A subsequent multivariate Cox regression analysis revealed that EVL expression, *T* classification, and *M* classification were all independent predictors of PACA patient postoperative OS duration (all *p* < 0.05) (Table 3).

## 4. Discussion

PACA is a deadly cancer type that is forecast to become the second leading cancer-associated cause of mortality in the future [11]. As such, novel diagnostic and prognostic biomarkers associated with this disease must be identified in an effort to improve patient treatment and survival outcomes. Prior research has shown that genes that are dysregulated in PACA may offer value as prognostic or diagnostic biomarkers for patients with this cancer type [12–16]. Plasma biomarkers are particularly attractive targets for patient diagnosis, staging, and monitoring as they can be assessed via a relatively noninvasive liquid biopsy approach. After being released from cells, RNA molecules form complexes with lipids that protect these RNAs from nuclease-mediated degradation [17–19]. In general, cancer patients exhibit higher levels of circulating RNA than do healthy individuals owing to the higher rates of tumor cell proliferation and apoptotic death in the former cohort [20]. As such, in the present study, we sought to identify candidate plasma mRNA biomarkers capable of predicting PACA patient survival outcomes.

We began by employing a bioinformatics approach to assess PACA-related mRNA expression profiles in the TCGA database as a means of detecting potential prognostic biomarkers in these cancer patients. However, mRNAs that are differentially expressed in tumor tissues may not necessarily be differentially expressed in patient plasma samples, given that normal tissues also contribute to plasma RNA profiles and have the potential to mask tumor-derived mRNA signals in circulation [21]. By comparing our TCGA findings to the results of a microarray analysis of PACA patient plasma samples, we identified just three prognosis-related DE mRNAs in these plasma samples: PTPN6, EVL, and SMAP2.

Through further validation experiments, we confirmed that EVL mRNA expression was decreased in PACA patient plasma samples relative to samples from healthy controls. Decreased EVL mRNA expression was associated with poor OS and with tumor pathological stage and was an independent predictor of PACA patient prognosis. EVL is an Ena/VASP (enabled/vasodilator-stimulated phosphoprotein) family member protein involved in actin cytoskeleton regulation [22, 23]. Alterations in cytoskeletal composition can influence cellular motility, ultimately driving or suppressing tumor cell invasion and migration. Mouneimne et al. suggested that EVL downregulation was capable of suppressing tumor cell migration and invasion in vitro and in vivo, and decreased EVL expression in human tumor cells has been shown to be associated with high invasive activity, increased protrusion, decreased contractility, and reduced adhesion [24]. Grady et al. found EVL to be commonly downregulated in human colorectal cancer through a mechanism associated with altered CpG methylation upstream of EVL [25]. Li et al. found EVL mRNA expression to be decreased in cervical cancer [26]. As such, we hypothesize that EVL downregulation in PACA patients promotes disease progression via driving tumor invasion and metastasis, ultimately leading to poor patient outcomes.

## 5. Conclusions

In summary, we validated the prognostic value of EVL in patient plasma samples, revealing that reduced plasma EVL expression is an independent predictor of PACA patient prognosis.

## Figures and Tables

**Table 1:** The expression of EVL and clinicopathologic features in 79 pancreatic cancer patients.

Characteristics	EVL		*p* value (*χ*2 test)
	Low expression	High expression	
Age			0.010
≦60	6	16	
>60	34	23	

Gender			0.556
Male	18	15	
Female	22	24	

*T* classification			0.417
I, II	21	24	
III, IV	19	15	

*N* classification			0.516
No	26	28	
Yes	14	11	

Metastasis			1.000
No	37	37	
Yes	3	2	

Clinical stage			0.260
I, II	31	34	
III, IV	9	5	

Pathological differentiation		0.007	
1, 2	19	30	
3, 4	21	9	

Vessel invasion			0.106
No	33	26	
Yes	7	13	

Nerve invasion			0.406
No	9	12	
Yes	31	27	

**Table 2:** Spearman analysis of the correlations between EVL and clinicopathological variables.

	EVL expression level	
Variables	Spearman correlation	*p* value
Age (year)	−0.290	0.009
Gender	0.066	0.562
*T* classification	−0.091	0.424
*N* classification	−0.073	0.522
Metastasis	−0.049	0.670
Clinical stage	−0.127	0.266
Pathological differentiation	−0.303	0.007
Venous invasion	0.182	0.108
Nervous invasion	−0.094	0.412

**Table 3:** Univariate and multivariate Cox regression analyses of prognostic parameters in pancreatic cancer patients.

	Univariate analysis		Multivariate analysis		
	*p* value	Regression coefficient (SE)	*p* value	Relative risk	95% confidence interval
*T* classification	0.009	2.065 (0.276)	0.037	1.795	1.036–3.110
Metastasis	0.002	5.374 (0.541)	0.001	6.785	2.253–20.428
EVL expression	0.010	0.508 (0.264)	0.010	0.491	0.286–0.841

## Data Availability

Part of the RNA sequence dataset in the findings was taken from the TCGA public database. Other data used to support the findings of this study are available from the corresponding author upon request.
